# Suppressing Photoinduced Charge Recombination via the Lorentz Force in a Photocatalytic System

**DOI:** 10.1002/advs.201901244

**Published:** 2019-07-22

**Authors:** Wenqiang Gao, Jibao Lu, Shan Zhang, Xiaofei Zhang, Zhongxuan Wang, Wei Qin, Jianjun Wang, Weijia Zhou, Hong Liu, Yuanhua Sang

**Affiliations:** ^1^ State Key Laboratory of Crystal Materials Shandong University Jinan 250100 P. R. China; ^2^ Shenzhen Institutes of Advanced Technology Chinese Academy of Sciences Shenzhen 518055 P. R. China; ^3^ Institute for Advanced Interdisciplinary Research (iAIR) University of Jinan Jinan 250022 P. R. China

**Keywords:** charge separation, Lorentz force, photocatalysis

## Abstract

Suppressing the recombination of photogenerated charges is one of the most important routes for enhancing the catalytic performance of semiconductor photocatalysts. In addition to the built‐in field produced by semiconductor heterostructures and the photo‐electrocatalysis realized by applying an external electrical potential to photocatalysts assembled on electrodes, other strategies are waiting to be scientifically explored and understood. In this work, a Lorentz force–assisted charge carrier separation enhancement strategy is reported to improve the photocatalytic efficiency by applying a magnetic field to a photocatalytic system. The photocatalytic efficiency can be improved by 26% just by placing a permanent magnet beneath the normal photocatalytic system without any additional power supply. The mechanism by which the Lorentz force acts oppositely on the photogenerated electrons and holes is introduced, resulting in the suppression of the photoinduced charge recombination. This work provides insights into the specific role of the Lorentz force in suppressing the recombination of electron–hole pairs in their initial photogenerated states. This suppression would increase the population of charge carriers that would subsequently be transported in the semiconductor. It is believed that this strategy based on magnetic effects will initiate a new way of thinking about photoinduced charge separation.

## 1 Introduction

Photocatalysis converts solar energy into highly active chemical energy, which can then be used for chemical synthesis or decomposition. In addition, photocatalysis provides an effective solution, based on solar energy conversion, for energy shortages and environmental crises.^1^ After nearly 40 years of investigation, researchers have developed approaches to design and synthesize high‐performance photocatalysts for different applications based on an understanding of the photocatalytic mechanisms. In general, photocatalytic efficiency is mainly limited by light absorption,^2^, ^3^ photoelectric conversion, and the utilization efficiency of photogenerated charge carriers. Generally, there are two categories of approaches to enhance the performance of a photocatalyst. One is to expand the photocatalytic active spectral range of semiconductors to increase the photogenerated charge carrier density, which has been extensively studied.^4^ The other is to improve the utilization efficiency of photogenerated charge carriers based on enhancing charge carrier separation.

One of the most important approaches to enhance the photoinduced carrier separation in semiconductors is the construction of different heterostructures, such as Schottky junctions,^5^ p–n junctions,^6^ type‐II heterostructures,^7^ and z‐scheme heterostructures,^8^ which are believed to play a key role at the material interfaces due to the generation of built‐in electric fields. These heterostructures have provided a practical means to design and construct high‐performance photocatalysts. However, heterostructures should be specifically designed for different semiconductors and for different applications. Moreover, the synthetic approaches for heterostructures are normally complicated and require good control, which limit their universal application to enhancing photocatalytic properties. The other approach for increasing photoinduced charge carrier separation is to apply an external electric bias to the photocatalyst by assembling the semiconductor on an electrode with a direct current supply.^9^ Thus, the applied bias can efficiently separate the photogenerated electrons and holes, resulting in highly efficient charge separation. However, charge separation relies on a continuous energy supply, which results in the high complexity of photocatalytic devices and higher costs. The magnetic field effect (MFE) technique has been used to investigate the spin–orbital coupling in carbon‐based semiconductor film devices.^10^ It is accepted that magnetic field affects the formation of relatively long‐lived spin‐1/2 electron–hole pair.^11^ The applications of magnetic field on silicon solar cell and organic–inorganic perovskite solar cell show a significantly improved photoelectron conversion efficiency.^12^ Similarly, the improvement of photoinduced carriers during the photocatalytic process might be achieved via the involving of magnetic field effect. Moreover, it is meaningful and interesting to study and discover new strategies for efficient charge carrier separation in powder photocatalysts.

The Lorentz force in a magnetic field, the force produced on a charge induced by the relative motion between the charge and the magnetic field, is defined as: $\vec{F}=q\left(\vec{V}\times \vec{B}\right)$, where *q* is the particle charge and $\vec{V}$ is the velocity of a particle moving in a magnetic field with a magnetic induction intensity ($\vec{B}$). According to the left‐hand rule, a moving charge in a magnetic field should experience a force vertical to the direction of movement in the magnetic field plane, which results in a deviation of the charge movement. Although the deviation effect of the Lorentz force on moving charges is well‐known, it has not been well‐discussed in photocatalytic processes.^13^ During the photocatalytic process, the electron–hole pairs generate in the semiconductor after light absorption. When the electron–hole pair moves together with the nanomaterial in a magnetic field, the electron and hole will experience opposite forces due to their opposite charges and deviate in opposite directions. Based on this mechanism, we proposed a magnetic field–enhanced photocatalytic process.

In the current work, a highly crystalline TiO_2_ nanobelt was selected as a model photocatalyst to study the mechanism of enhancement of the photocatalytic properties in a static magnetic field. The nonmagnetic property of TiO_2_ should constrain the interactions of the photogenerated charges and magnetic field. The results of photocatalytic methyl orange (MO) degradation showed an improvement in the photocatalytic efficiency just by placing a permanent magnet beneath the photocatalytic reactor. Further tests were performed to discuss the interactions between the photocatalyst and the magnetic field and its effects on the photocatalytic enhancement. A mechanism for the separation of the initial photogenerated charges was proposed and discussed. This work represents a new facile strategy to enhance the photocatalytic process, which may be suitable for a number of semiconductor photocatalysts.

## 2 Results and Discussion

**Figure 1:**
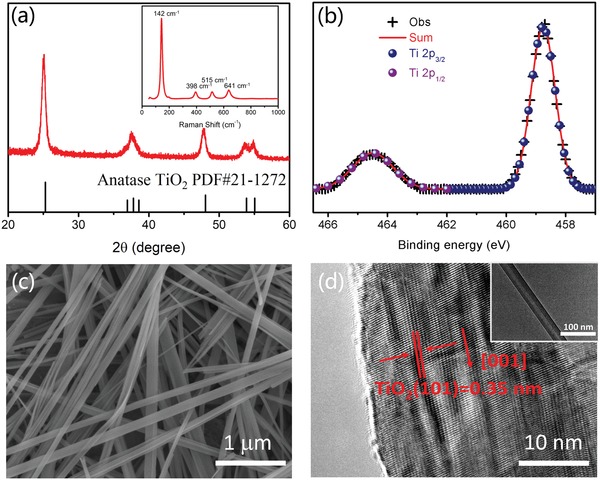
a) XRD pattern and Raman spectrum (inset) of the TiO_2_ nanobelts. b) High‐resolution Ti 2p XPS spectrum of TiO_2_. c) Typical SEM image of the as‐obtained TiO_2_ nanobelts. d) High‐resolution TEM image and TEM image (inset) of the TiO_2_ nanobelts.

The structure and morphology of the TiO_2_ nanobelts were analyzed according to their X‐ray diffraction (XRD) pattern, Raman spectrum, X‐ray photoelectron spectroscopy (XPS) spectrum, and scanning electron microscopy (SEM) and high‐resolution transmission electron microscopy (HRTEM) images (**Figure** **1**). The XRD pattern shown in Figure 1a indicates the pure anatase TiO_2_ phase of the as‐obtained TiO_2_ nanobelts (JCPDS No. 21‐1272). The Raman spectrum confirms the anatase structure of the TiO_2_ nanobelts (inset of Figure 1a). The XPS survey spectrum of the TiO_2_ nanobelts confirms the presence of only Ti and O (Figure S3, Supporting Information). Analysis of the high‐resolution Ti 2p XPS spectrum shows that only the characteristic peaks attributable to Ti^4+^ can be detected (Figure 1b). This indicates that there are few oxygen defects and no other valence states of Ti in the as‐obtained TiO_2_ nanobelts. The TiO_2_ nanobelts are 50–200 nm in width and up to tens of micrometers in length (Figure 1c). The surface of the TiO_2_ nanobelts was very clean and smooth (Figure 1c and inset of d). The lattice image of the as‐obtained TiO_2_ nanobelts shows an interplanar spacing of 0.35 nm (Figure 1d), which corresponds to the (101) plane and a [001] growth direction for the nanobelts. These results further confirm the good crystalline structure. Notably, the high crystallization would reduce photoinduced charge carrier recombination during transport. Therefore, we chose these well‐crystalline TiO_2_ nanobelts as an ideal model for investigating the effect of a magnetic field on photoinduced charge carrier separation.

**Figure 2:**
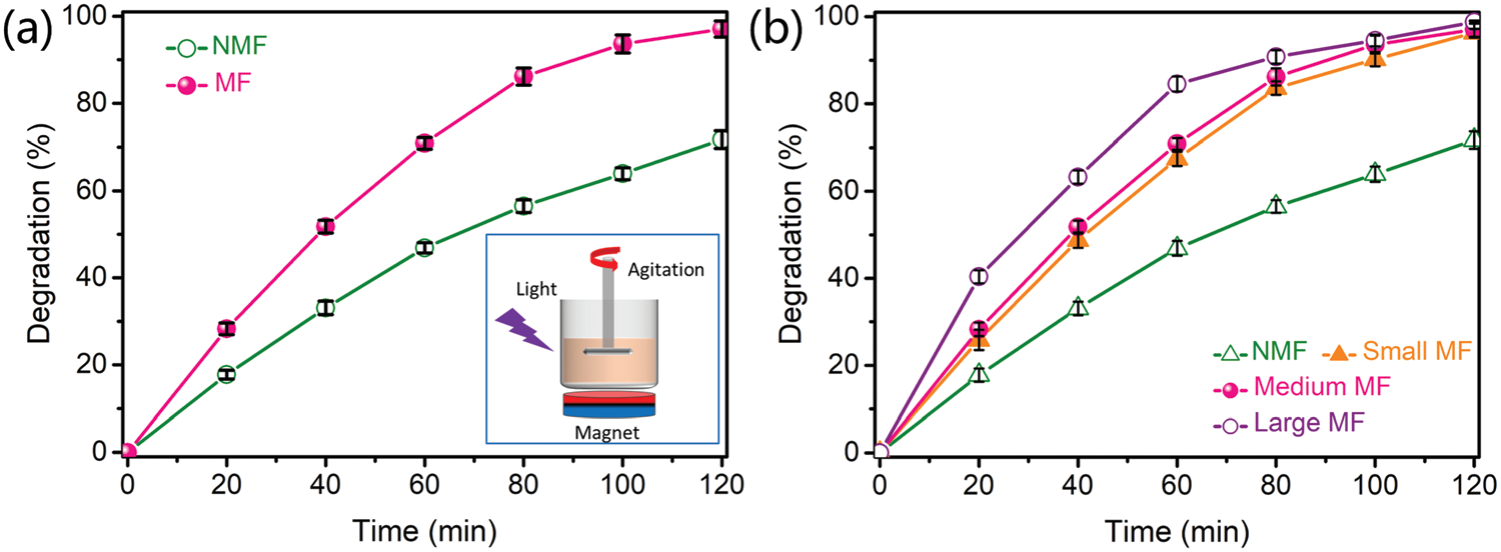
a) Photocatalytic degradation of MO with no magnetic field (NMF) or with a magnetic field (MF) in the presence of TiO_2_ nanobelts under 20 mW cm^−2^ UV illumination. The inset is a schematic diagram of the magnetic field photocatalytic setup. b) Photocatalytic degradation of MO with magnetic fields of different magnetic induction intensities.

For the powder catalyst, the interaction between the photocatalyst and the magnetic field is difficult to detect directly. As is well‐known, the photocatalytic activity would be limited by the charge separation of the catalyst. Thus, the photocatalytic degradation of MO with the TiO_2_ nanobelts was performed with and without a magnetic field (MF and no magnetic field (NMF)) to indirectly evaluate the effect of the magnetic field on the photocatalyst (**Figure** **2**). Figure 2a shows the photocatalytic performance of the TiO_2_ nanobelts under the MF and NMF condition by using the magnetic photocatalyst setup (inset of Figure 2a). The magnetic induction intensities at the surface of the permanent magnet were at three levels: small (580 Gauss), medium (810 Gauss), and large (1400 Gauss). Agitation of the suspension provided the relative movement between the photocatalysts and the magnetic field. For the general photocatalytic reaction device, the photodegradation rate of MO was 71% after 2 h of UV illumination. Notably, the photodegradation rate of MO after 2 h was 97% for the medium MF (810 Gauss) under the same reaction conditions. The inclusion of an MF resulted in an ≈26% improvement in the photocatalytic properties, which indicates that the existence of a permanent magnetic field plays a remarkable role in improving the photocatalytic efficiency.

To understand the cause of the photocatalytic improvement in an MF, the photocatalytic activities of the TiO_2_ nanobelts were evaluated at different MF intensities (Figure 2b). Three different intensities of the magnetic field were produced by different arrangements of the Nd_2_Fe_14_B magnets, which were defined as small, medium, and large magnetic fields with intensities of 580, 810, and 1400 Gauss on the surface of the magnets, respectively (Figures S4–S6 in the Supporting Information show the detailed testing process). At the same stirring speed (600 rpm), the degradation rates of MO with the TiO_2_ nanobelts were 46%, 67%, 70%, and 84% after UV illumination for 60 min under the conditions of NMF, small MF, medium MF, and large MF, respectively. The results demonstrate a noticeable enhancement in the photocatalytic performance as the magnetic induction intensity $\left(\vec{B}\right)$ increases.

The stirring speed relates to the relative movement between the photocatalyst and the magnetic field. Therefore, the photocatalytic degradation rates of MO improved at higher stirring speeds (Figure S7, Supporting Information). This confirms the effect of relative movement on the interaction between the photocatalyst and the magnetic field. A more intuitive comparison of the photocatalytic performance with UV irradiation times ranging from 20 to 80 min in various magnetic fields and with various stirring speeds confirms the improvement of the photocatalytic efficiency with increasing magnetic induction intensity and stirring speed (Figure S8, Supporting Information). Notably, the photocatalytic degradation rate of MO with the TiO_2_ nanobelts follows first‐order reaction dynamics under both various magnetic fields and various stirring speeds (Figure S9, Supporting Information). This indicates that the magnetic field strength and the stirring speed do not influence the catalytic process.

The results confirm that the magnetic induction intensity $\left(\vec{B}\right)$ and stirring speed have significant effects on the photocatalytic performance. The stirring of the suspension results in the movement of the TiO_2_ nanobelts $\left(\vec{V}\right)$ along with the suspension in an MF. Therefore, increasing the magnetic induction intensity $\left(\vec{B}\right)$ and movement speed of the TiO_2_ nanobelts $\left(\vec{V}\right)$ results in a larger Lorentz force acting on the moving charges according to the equation for a Lorentz force produced in a magnetic field: $\vec{F}=q\left(\vec{V}\times \vec{B}\right)$, thereby improving the deviation of the electrons and holes when moving in a magnetic field and leading to better separation of the charge carriers. According to the analysis above, a preliminary conclusion can be drawn that the Lorentz force plays an important role in improving the photocatalytic activity of the TiO_2_ nanobelts by influencing the photogenerated charge carriers.

The photocatalytic performance of the TiO_2_ nanobelts under visible light with an MF and NMF are almost the same, indicating that without the charge carriers produced by photoelectric conversion, the Lorentz force cannot enhance the photocatalytic process (Figure S10, Supporting Information). Moreover, with the inclusion of a hole‐capture agent, the degradation rates of MO were quite similar and low with an MF and NMF. In contrast, with the addition of an electron‐capture agent, the degradation rates were significantly improved, which further confirms the importance of charge separation for improving the photocatalytic process. The further improvement of the photocatalytic performance of the TiO_2_ nanobelts with an MF in the presence of an electron‐capture agent confirms the improved charge separation in an MF (details in Figures S11 and S12, Supporting Information).

**Figure 3:**
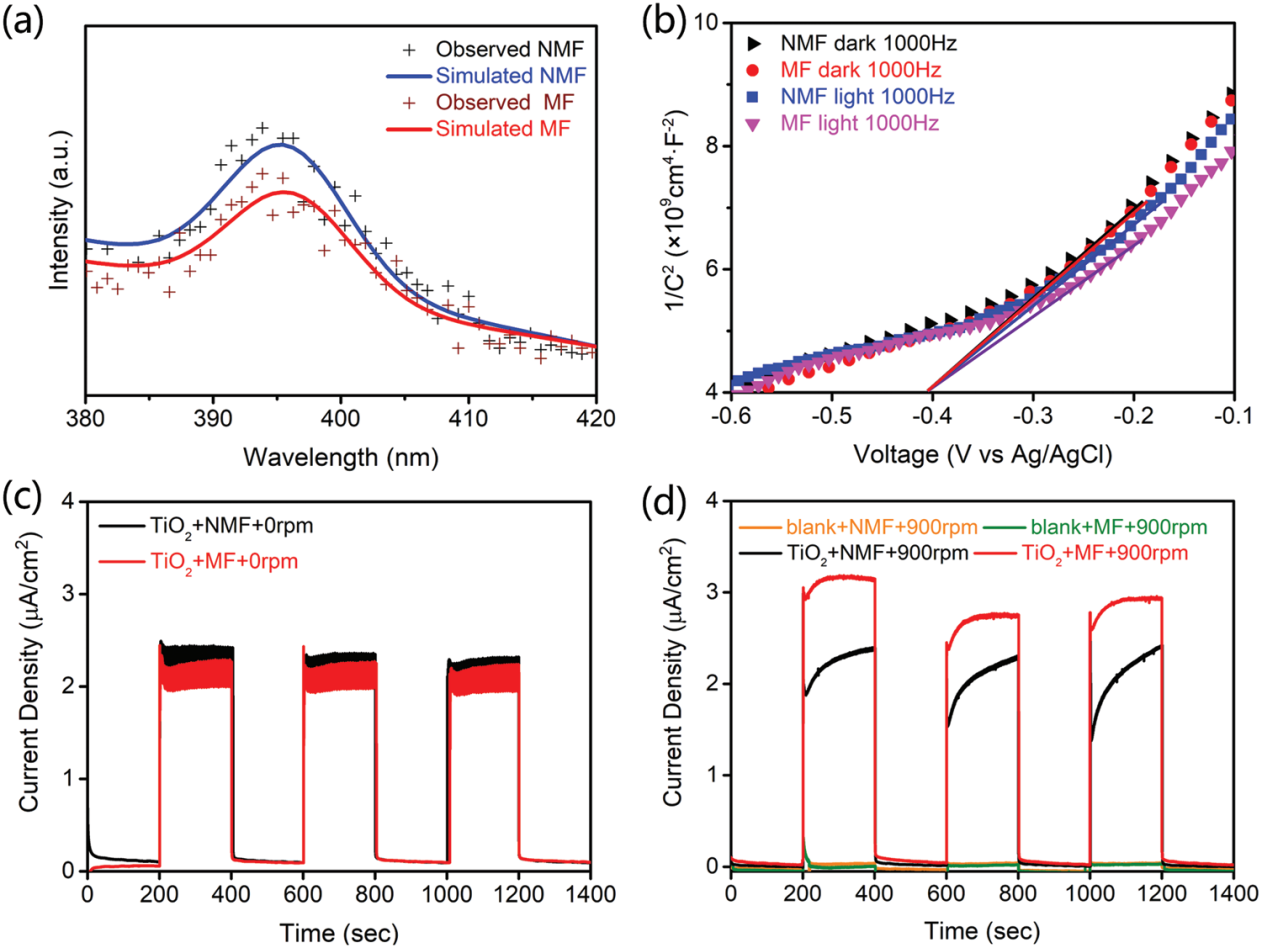
a) Photoluminescent spectra excited at 365 nm under NMF and MF conditions. b) Mott–Schottky plots of TiO_2_ under NMF and MF conditions in the dark and illuminated with UV light. The electrolyte is 0.1 m Na_2_SO_4_. c) The *I*–*t* curves of the TiO_2_ nanobelts without rotation under NMF and MF conditions. d) The *I*–*t* curves of the TiO_2_ nanobelts and blank electrode with on–off switching of the illumination and a 900 rpm electrode rotation under NMF and MF conditions.

To study the photoinduced charge carrier separation properties, the photoluminescent spectra, Mott–Schottky plots, and the *I*–*t* curves with on–off switching of the illumination are shown in **Figure** **3**. The photoluminescence (PL) intensity of the TiO_2_ nanobelts under MF conditions is lower than that under NMF conditions (Figure 3a). TiO_2_ materials always possess a relatively high PL intensity, which corresponds to the high rate of photoinduced charge recombination. The lower intensity under MF condition indicates that the interaction between the TiO_2_ nanobelts and the magnetic field reduces the charge recombination rate.

Mott–Schottky plots describe the reciprocal of the square of the capacitance versus the potential difference between a semiconductor and an electrolyte.^14^ For the results of the TiO_2_ nanobelts, the negative slope confirms the n‐type semiconductor property of the material (Figure 3b), indicating that the photoinduced electrons are the dominant charge carriers. The analyzed flat‐band potentials of the TiO_2_ nanobelts are similar and independent of the light or/and magnetic field conditions. According to the function, slope = 2(*εε*_0_ *A* ^2^ *eN*_D_)^−1^, where ε and ε_0_ are dielectric constants, *A* is the electrode area, and *N*_D_ is the dopant density in the semiconductor, which can be assigned to the charge carrier density,^15^ a smaller slope indicates a higher carrier density. The slopes barely change in the dark under NMF and MF conditions, which indicates that the magnetic field does not influence the intrinsic charge carrier density. With light illumination, the slope is smaller than that in the dark under NMF conditions. This is consistent with the generation of photoinduced charges, which results in a higher charge density. When illumination is combined with an MF, the slope is further decreased. This indicates an even higher charge carrier density induced by the magnetic field. The *N*_D_ calculated under NMF condition with light illumination is 2.58 × 10^17^ n cm^−3^, while the value increases to 3.21 × 10^17^ n cm^−3^ when an MF condition is applied. This 24% improvement in the charge carrier density is consistent with the 26% improvement in the photocatalytic degradation rate. This implies that the photocatalytic improvement mostly comes from the increase in charge carriers induced by the magnetic field.

Applying an additional voltage to the photoelectrode would surely improve the photogenerated charge carrier transport. The current density is directly related to the photoinduced charge carrier density. Therefore, the *I*–*t* curves were recorded under NMF and MF conditions to analyze the changes in the photoinduced charge carrier density. As shown in Figure 3c, without rotation of the electrode, the photocurrent densities of the TiO_2_ nanobelts are both 2 µA cm^−2^ at a bias of 0.2 V under both NMF and MF conditions. Based on the requirements for a Lorenz force in a magnetic field, without rotation of the electrode, there is no interaction between the photocatalyst and the magnetic field. Therefore, the *I*–*t* curves under NMF and MF conditions are the same when the electrode is not rotated. With the rotation of the electrode at 900 rpm, the photocurrent density is still ≈2 µA cm^−2^ under NMF conditions; however, the photocurrent density increases to 2.7 µA cm^−2^ under MF conditions (Figure 3d). The requirement of rotation is quite different from solar cell investigation with static magnetic field where the existence of spin‐pair species is the precondition.^12^ The photocurrent densities of the electrode prepared without TiO_2_ nanobelts are only ≈0.1 µA cm^−2^ at a rotation of 900 rpm. Therefore, the ≈35% increase in the photocurrent density can be assigned to the interaction, which should be the Lorentz force in a magnetic field, between the photocatalyst and magnetic field realized via the rotation of the electrode in a magnetic field. The increase in the photocurrent density is ≈10% larger than the increase in the photocatalytic degradation of MO (Figure 2a). That is, most of the newly separated charges under MF conditions can be transported and detected in the *I*–*t* curve. However, some of these charges might recombine during transport in the TiO_2_ nanobelt suspension during the photocatalytic process. The open‐circuit potentials (OCPs) under on–off switching of the light illumination were recorded under NMF and MF conditions (Figure S13, Supporting Information). An enhancement in the OCP of 3 mV in an MF during rotation indicates the increase in the number of photogenerated charge carriers.

**Figure 4:**
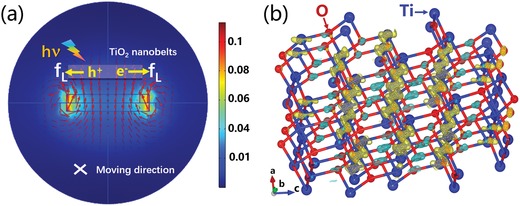
a) Schematic diagram of the interaction between the magnetic field and the moving photocatalyst under illumination. b) Charge density differences of the (101) TiO_2_ surface with/without an external electric field (0.01 V Å^−1^). The charge density difference is defined as *C*_E_ − *C*_noE_, where *C*_E_ and *C*_noE_ denote the charge density of the system with and without an external electric field, respectively. The (1 × 1) supercells used in the calculation are replicated in the in‐plane direction for ease of viewing.

The interaction between the photocatalyst and the magnetic field is difficult to describe based on first‐principles theory, and an equivalent interaction was used instead. As shown in **Figure** **4**a, when illuminated, photoinduced electrons and holes form in the TiO_2_ photocatalyst. As the charged catalyst moves along the liquid flow, crossing the magnetic induction lines, the Lorentz force acts on the electrons and holes, driving them in opposite directions. Comparing the Lorentz force of $\vec{F}=q\left(\vec{V}\times \vec{B}\right)$ with the electric force of ${\vec{F}}_{E}={\vec{E}}_{q}$, the effects of the magnetic field on the charge carriers can be discussed by referring to the electric field. Briefly, the ${\vec{F}}_{L}$ was ≈0.01*q* in the current study. Therefore, the electric field intensity $\vec{E}$ was set to 0.01 V m^−1^ (Figures S14 and S15, Supporting Information). It can be seen from the charge density differences (Figure 4b) that an external electric field leads to a spatial charge transfer between orbitals of different atoms (here, from O 2p to the Ti 3d orbitals). It can be intuitively imagined that this effect facilitates the spatial separation of the photogenerated electron–hole pairs, given that the electric field forces acting on the electrons and holes are in opposite directions. For an applied magnetic field, the Lorentz force acting on the photogenerated electrons and holes are also opposite in direction, thus promoting their spatial separation and inhibiting recombination.

**Figure 5:**
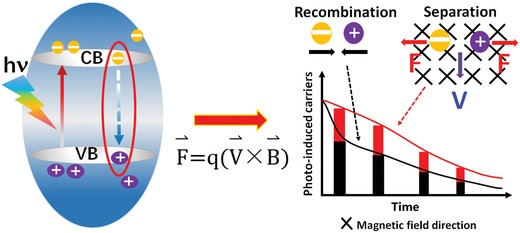
Schematic illustration of the proposed influence of the magnetic field on photoinduced charge carrier separation in the TiO_2_ nanobelts.

The influence of the magnetic field on photoinduced charge carrier separation is proposed and illustrated in **Figure** **5**. For typical cycle of the photoinduced charge carriers in a semiconductor, when a photon is absorbed by the semiconductor, a photogenerated electron and hole form at the same time, which is known as an “electron–hole pair.” The electron and hole can easily recombine because the formed excited state obeys the conservation laws for energy and momentum. Charge recombination releases energy as light or heat and results in the rapid decay of the photoluminescence.^16^ Therefore, the density of the initial photogenerated charge carriers that participate in charge transport is limited. A potential or force to drive the positive and negative carriers in opposite directions would significantly enhance the charge carrier density. In fact, numerous photocatalytic enhancement approaches have been based on this mechanism, such as built‐in field construction, Z‐scheme heterostructures, and photo‐electrocatalytic approaches.^17^ In this work, a magnetic field was present during photocatalysis. According to the left‐hand rule, both the moving electrons and holes in the magnetic field experience a Lorentz force vertical to their direction of movement. Moreover, the forces acting on the negative electron and positive hole of the electron–hole pair are in opposite directions, which results in the deviation of the electron and hole in opposite directions. Therefore, in a magnetic field, the photoinduced electron–hole pairs in moving semiconductor particles can be separated, even at the initial generation of an electron–hole pair. The expected fluorescence lifetime spectra are assumed to illustrate the effect of the Lorentz force on the photoinduced charge carrier separation (Figure 5). Charge carrier recombination is initially rapid, producing a fast decay rate in the PL spectrum. The involvement of the Lorentz force prevents charge carrier recombination, which would slow the decay rate of the PL spectrum. This means that more charge carriers can participate in charge transport, resulting in more charge carriers participating in the photocatalytic process. Thus, the magnetic field can enhance the photocatalytic degradation of MO in a photocatalyst suspension under vigorous stirring. However, it should be noted that the nanobelts are rotating along the flow during the photocatalytic process. It means that the charges moving along the nanobelt might be turned over repeatedly, resulting in a long transport path. Therefore, the Lorentz force does not benefit the charge transport, but influences the charge recombination at the first stage of the generation.

## 3 Conclusions

In summary, the effect of the magnetic field on the photocatalytic properties of TiO_2_ nanobelts was studied. Highly crystalline TiO_2_ nanobelts with few defects were used as a model photocatalyst to discuss the improvement of the photocatalytic properties in a magnetic field. Briefly, the Lorentz force can act on the photoinduced charge carriers as the photocatalyst moves in a magnetic field. The Lorentz force suppresses charge recombination of the initial photogenerated charge carries, when charge recombination is severe. This results in more charge carriers being available for transport in the photocatalyst, and thus, more active charge carriers are available for the photocatalytic process. A 26% improvement of photocatalytic degradation of MO was achieved via placing a permanent magnet beneath the photocatalytic reactor only. This work provides new insights into the effect of a magnetic field on photogenerated charge carrier separation. With the proper design, an external magnetic field is expected to be widely used in the field of catalysis to improve the catalytic performance.

## 4 Experimental Section

*Materials*: All the chemicals in this work were of analytic grade and commercially available. TiO_2_ nanoparticles (P25) were purchased from Evonik‐Degussa Specialty Chemicals (Shanghai) Co., Ltd., and sodium hydroxide (NaOH), potassium bromate (KBrO), sodium oxalate (Na_2_C_2_O_4_), hydrochloric acid (HCl), ethanol, and methyl orange were purchased from Sinopharm Chemical Reagent Co., Ltd. All the chemicals were used without further purification.

*Preparation of the TiO_2_ Nanobelts*: To study the separation process of the photogenerated charge carriers and avoid unexpected factors, crystalline TiO_2_ nanobelts were used. The TiO_2_ nanobelts were synthesized via a hydrothermal method followed by postcalcination.^18^, ^19^ Typically, P25 (0.2 g) was dissolved in a 10 m NaOH solution (40 mL). After stirring and ultrasonic treatment, the homogeneous P25 suspension was transferred to a 50 mL Teflon‐lined autoclave and heated at 200 °C for 72 h. After washing thoroughly with deionized water, the obtained product was dispersed in a 0.1 m HCl solution for 24 h to obtain H_2_Ti_3_O_7_ nanobelts. After washing thoroughly with deionized water and alcohol, the precursor was calcined at 600 °C for 2 h in air. The TiO_2_ nanobelts were thus obtained.

*Characterization*: X‐ray diffraction of the sample was performed on a powder X‐ray diffractometer (Cu Kα, λ = 0.15406 nm, Bruker D8 Advance, Germany) to confirm the purity of the structure. The morphology of the as‐obtained sample was investigated by field‐emission scanning electron microscopy (FESEM, Hitachi S‐4800, Japan). The morphology, especially for the lattice image of the sample, was studied via high‐resolution transmission electron microscopy (JEOL JEM 2100 microscope, Japan). X‐ray photoelectron spectroscopy spectra were recorded on an ESCALAB 250 instrument to discuss the defects in the as‐obtained sample. The photoluminescent spectra of TiO_2_ were recorded with and without the influence of a magnetic field. The equipment was built in the lab from a fiber spectrograph (NOVA‐EX, 320‐1113 nm, Shanghai Ideaoptics, China). The excitation wavelength was 365 nm.

*Photocatalytic Activity Evaluation*: The photocatalytic activities of the samples were evaluated in terms of their degradation of methyl orange (10 mg L^−1^) in water with and without a magnetic field. Typically, without a magnetic field, TiO_2_ nanobelts (50 mg) were dispersed in an MO solution (100 mL) with stirring. The adsorption–desorption equilibrium of MO on the surface of the catalysts was reached after 120 min, and the suspension was then illuminated by a 40 W UV lamp (20 mW cm^−2^). The residual MO concentration in the supernatant was analyzed by UV–vis spectrophotometry (UV‐2102PC) at certain intervals. The error bars were obtained from four repeated runs for all the samples. Under various magnetic fields (Figure S1, Supporting Information), the degradation of MO was studied following the same procedure. The various magnetic fields were produced by Nd_2_Fe_14_B magnets. The magnets were 50 mm in diameter with various thicknesses. The thin magnet (3 mm in thickness) possessed a magnetic induction intensity of 580 Gauss, which was defined as the small magnetic field in this work. The thick magnet (10 mm in thickness) showed a magnetic induction intensity of 810 Gauss, which was defined as the medium magnetic field. Three thick magnets installed in series, with an intensity of 1400 Gauss, was defined as the large magnetic field. The effect of the stirring speed on the photocatalytic activity in a magnetic field was also studied. Moreover, hole‐capture or electron‐capture agents were also added during the degradation of MO to further evaluate the photocatalytic activity with and without the magnetic fields.

*Photoelectrochemical Tests*: The photoelectrochemical (PEC) performance was analyzed with an electrochemical workstation (CHI660C Instruments) using a standard three‐electrode cell in a 1 m NaOH aqueous solution. A Ag/AgCl electrode (saturated KCl) and Pt electrode were used as the reference electrode and counter electrode, respectively. Suspensions of the catalysts in a mixture of ethanol (4 mL) and Nafion (30 µL) were well‐dispersed at a concentration of 4 mg mL^−1^. The suspensions (10 µL) were dropcast onto rotating disk electrodes with working areas of 0.19 cm^2^. With and without a magnetic field, the *I*–*t* curves were recorded under the illumination of a UV lamp (50 mW cm^−2^) with light on–off intervals of 200 s and an applied bias of 0.2 V. The rotating disk electrodes were rotated at 0 and 900 rpm. The open‐circuit voltages were also recorded with light on–off intervals of 400 s. Electrodes on F‐doped tin oxide coated glass were also prepared with the suspensions. A new apparatus was designed to realize the interaction between the static photocatalyst and magnetic field (Figure S2, Supporting Information). Based on the newly observed interactions, the Mott–Schottky plots of TiO_2_ with and without a magnetic field were recorded at 1000 Hz under illuminated UV light. The electrolyte was 0.1 m Na_2_SO_4_. The carrier densities in the TiO_2_ nanobelts under various conditions were calculated.

*First‐Principles Calculations*: The first‐principles calculations were performed using the Vienna Ab initio Simulation Package (VASP). The calculations were based on plane‐wave Vanderbilt ultrasoft pseudopotentials.^20^ The generalized gradient approximation (GGA) with the Perdew–Burke–Ernzerhof (PBE) scheme was chosen to discuss the exchange–correlation potential.^21^, ^22^ To analyze the conditions of the different facets of TiO_2_, the (101), (001), and (100) surfaces of TiO_2_ with 1 × 1 surface cells consisting of 30, 18, and 7 atomic layers, respectively, were built. A 20 Å thick vacuum layer was used for all the surfaces to isolate the slabs. The sets of the atomic layers and vacuum layer of the system converged for the calculation of the energy well. The Brillouin zones of the (101), (001), and (100) surfaces were sampled at 7 × 10 × 1, 10 × 10 × 1, and 9 × 4 × 1 Γ‐centered k‐points, respectively. The electronic wave functions were expanded with plane waves with a cut‐off of 500 eV. The criterion for the relaxation of the structures was that all atomic‐force components were smaller than 10^−2^ eV Å^−1^. The tetrahedron method with Blöchl corrections was used to calculate the total and projected densities of states (DOSs and PDOSs) of the systems after geometry optimization. There is no proper strategy for applying a magnetic effect on a semiconductor. Because the calculations were used to discuss the charge carrier processes, the magnetic effect on the charges would be equivalent to that of an electric field on the charges. Electric fields normal to the slabs^23^, ^24^ were applied to model the effect of an external electric field on the electronic structures of the slabs. For simplicity, the structures with external electric fields were constrained to those optimized without the electric field.

## Conflict of Interest

The authors declare no conflict of interest.

## Supporting information

Supplementary
