# Supplementary material for: Suppressing Photoinduced Charge Recombination via the Lorentz Force in a Photocatalytic System
Source: Adv Sci (Weinh). 2019 Jul 22;6(18):1901244. doi: 10.1002/advs.201901244 (PMC6755512; doi:10.1002/advs.201901244)
Supplement: Supplementary file 1 — Supplementary [file ADVS-6-1901244-s001.pdf]

Copyright WILEY-VCH Verlag GmbH & Co. KGaA, 69469 Weinheim, Germany, 2019.

ADVANCED SCIENCE
Open Access

Supporting Information

for *Adv. Sci.,* DOI: 10.1002/advs.201901244

Suppressing Photoinduced Charge Recombination via the Lorentz Force in a Photocatalytic System

Wenqiang Gao, Jibao Lu, Shan Zhang, Xiaofei Zhang, Zhongxuan Wang, Wei Qin, Jianjun Wang, Weijia Zhou, Hong Liu,* and Yuanhua Sang**

Supporting Information of

Suppressing photo-induced charge recombination via Lorentz force in photocatalytic system

Wenqiang Gao, Jibao Lu, Shan Zhang, Xiaofei Zhang, Zhongxuan Wang, Wei Qin, Jianjun Wang, Weijia Zhou, Hong Liu* and Yuanhua Sang**

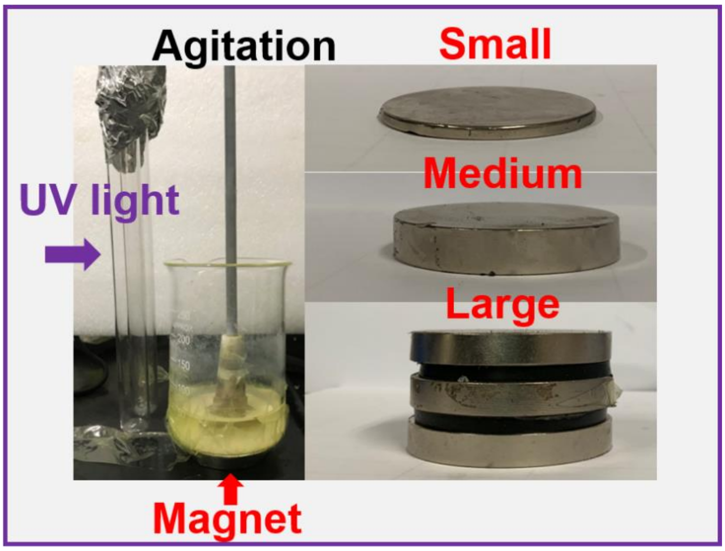

Figure S1. Photocatalytic degradation device and the different intensity of the magnets (small, medium, large).

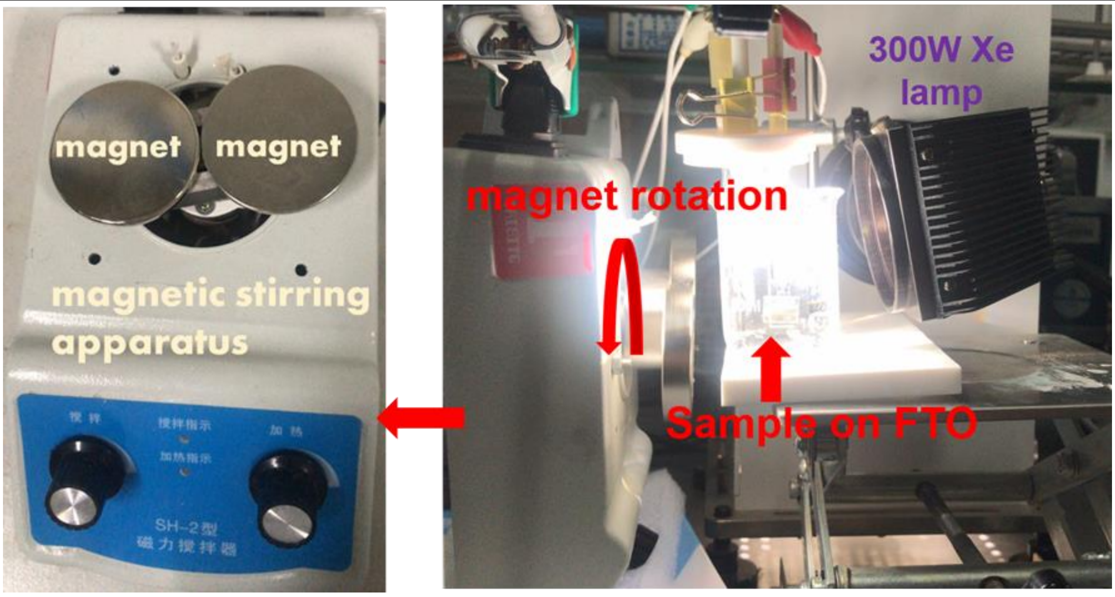

Figure S2. The new designed apparatus to realize the interaction between static photocatalyst and magnetic field.

As shown in Figure S2, we designed a new apparatus to realize the interaction between static photocatalyst and magnetic field. The photo (left) shows the system of the Mott–Schottky plots measurement.

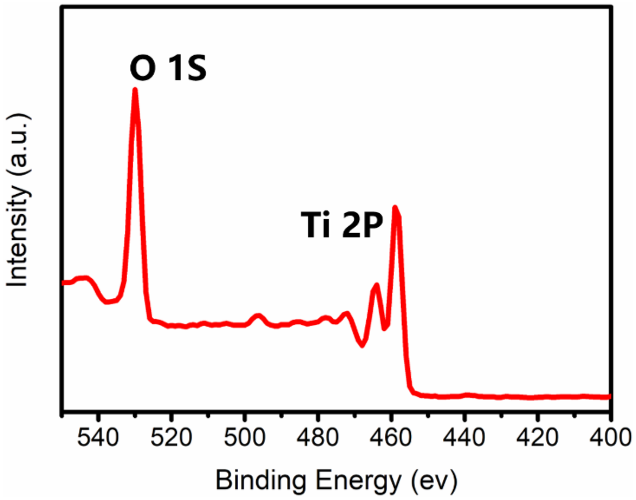

Figure S3. The survey XPS of the TiO_2 nanobelts.

Figure S3 shows the elemental composition and chemical states of the elements on the surface of the as-synthesized TiO_2 nanobelts. From the XPS survey of the TiO_2 nanobelts, the peaks refer to Ti and O can be identified besides of the C signal.

We made use of Gauss meter to measure the magnetic induction intensity of each point in space through establishing a simple spatial marker point above the magnet. As shown in Figure S4, we set up test points every two centimeters along the vertical direction of the magnet marked as 1, 2, 3 and 4 and the horizontal diameter is 3.5 cm. Meanwhile, at each point perpendicular to the magnet, three horizontal diameter ranges (3.5 cm, 4.5 cm and 6.5 cm) were selected horizontally to test its magnetic induction intensity. Detailed magnetic induction test data are shown in Figure S4-S6.

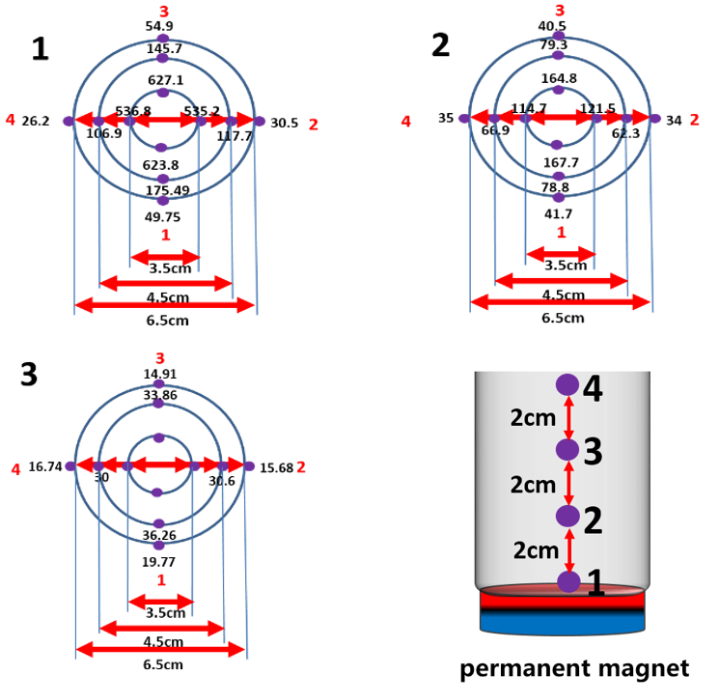

Figure S4. The intensity of the small magnetic field (one NdFeB magnet, thickness (T) 3 mm, diameter (D) 50 mm) by Gauss meter measure.

Figure S4 exhibited the magnetic induction intensity of a magnet with diameter of 50 mm and thickness of 3 mm in space. Moreover, in the plane with a vertical height of 6cm, the magnetic induction intensity was already zero, so it is not marked in Figure S4.

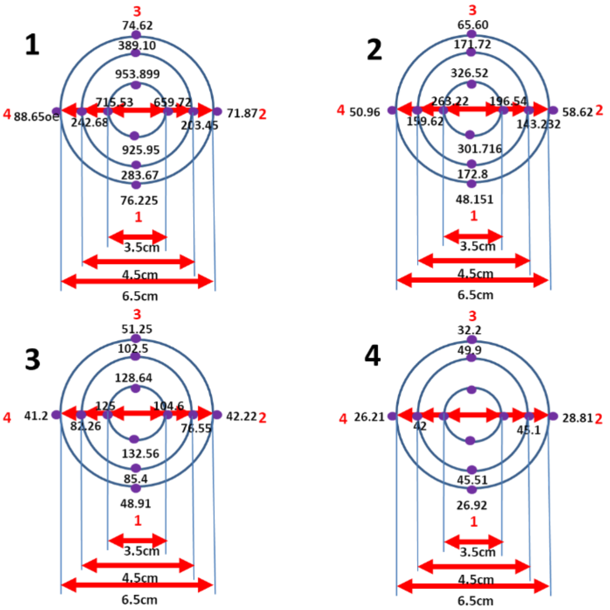

Figure S5. The intensity of the medium magnetic field (one NdFeB magnet, thickness (T) 10 mm, diameter (D) 50 mm) by Gauss meter measure.

Figure S5 exhibited the magnetic induction intensity of a magnet with diameter of 50 mm and thickness of 10 mm in space.

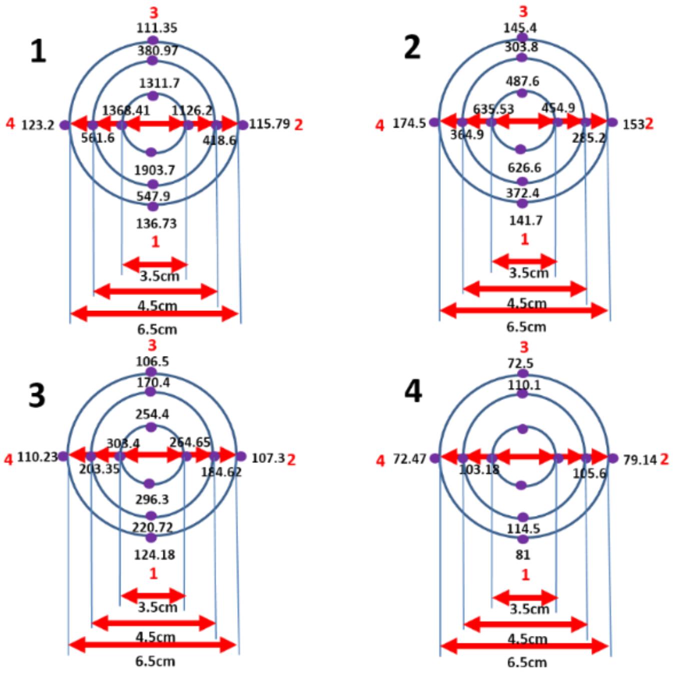

Figure S6. The intensity of the large magnetic field (three NdFeB magnets, thickness (T)) 10 mm, diameter (D) 50 mm) by Gauss meter measure.

Figure S6 exhibited the magnetic induction intensity of three stacked magnets with diameter of 50 mm and thickness of 10 mm in space.

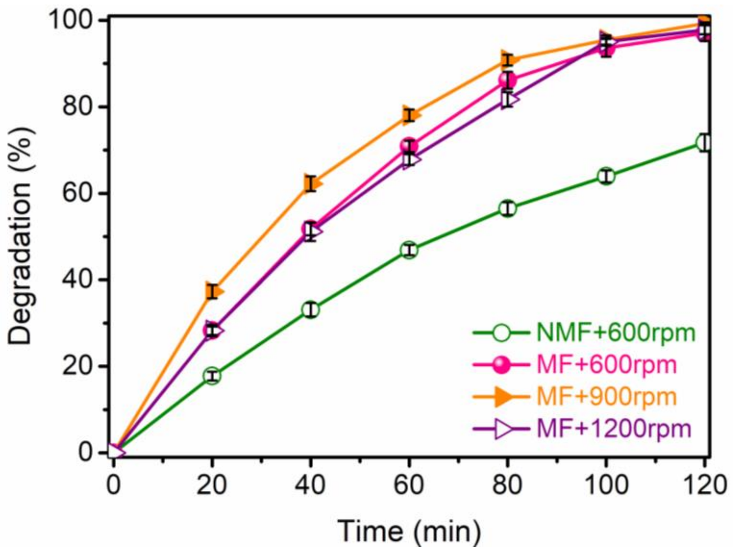

Figure S7. Photocatalytic degradation of MO with different stirring speeds in the same magnetic field intensity.

With the same magnetic field (medium MF), Figure S7 shows the photocatalytic activities of TiO_2 nanobelts at various stirring speeds. The degradation rates of MO are 46%, 70%, 78% and 67% under 60 min illumination at the stirring speeds of 0 rpm, 600 rpm, 900 rpm and 1200 rpm respectively. The results indicate a remarkable enhancement of photocatalytic performance with the increase of the stirring speed till 900 rpm. However, with a higher-speed stirring (1200 rpm), the raise of the liquid level would make the suspension too far from the magnet, and most of suspension attaches on the wall of the baker, which would result in a weak effect of magnetic field. The spatial distribution of the magnetic field measured by a gauss meter also proves this, the farther from the permanent magnet, the weaker the magnetic field would be. It indicates that the magnitude of the Lorentz force is related to the linear velocity and the magnetic induction intensity of the position of the charge. Therefore, the improvement of photocatalysis significantly is receded under an over speed stirring (1200 rpm).

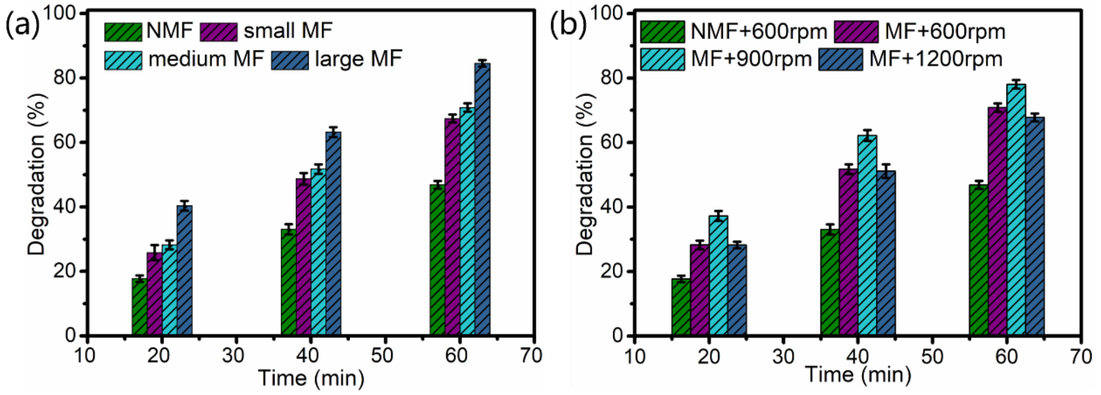

Figure S8. (a) Photocatalytic degradation of MO with various magnetic fields during 20 to 60 min. (b) Various rotation speeds under the same magnetic field intensity.

The histogram was used to further intuitively compare the degradation efficiency under different magnetic induction intensities and different rotating speeds, which was shown in

Figure S8. The degradation rates of TiO_2 nanobelts at the large magnetic field were 40%, 63%, 84% and 90% at 20, 40, 60, and 80 min respectively, higher than that at other low magnetic field intensities shown in Figure S8 (a). For different rotate speeds, the photocatalytic degradation rates at 900 rpm were 37%, 62%, 78% and 90% at 20, 40, 60 and 80 min respectively, which is higher than that of 1200 rpm (28%, 51%, 67%, and 81% at 20, 40, 60 and 80 min respectively), which is due to the larger disturbance at high speed (1200 rpm) from Figure S8 (b).

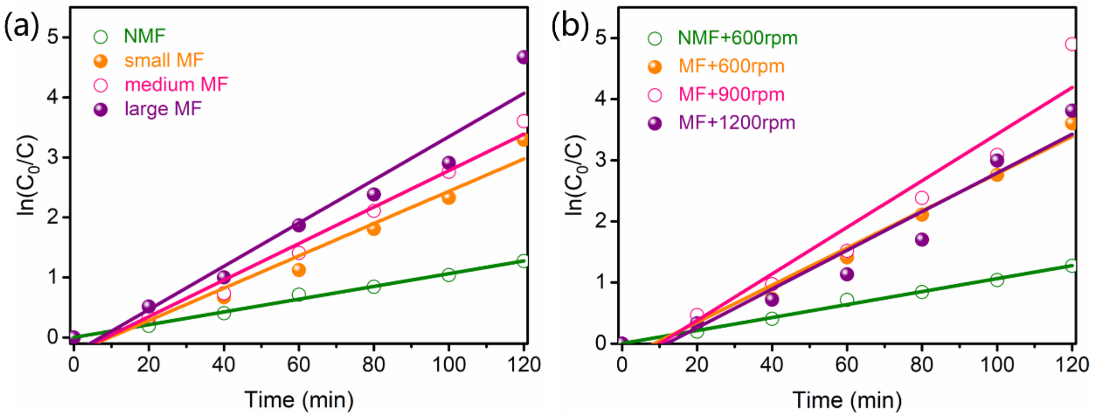

Figure S9. (a) ln (C_0/C) as a function of UV irradiation time in the presence of the TiO_2 nanobelts with different magnetic field intensities. (b) ln (C_0/C) as a function of UV irradiation time in the presence of the TiO_2 nanobelts with various rotation speeds under the same magnetic field intensity.

As shown in Figure S9 (a), the photocatalytic degradation in various magnetic fields follows the first-order reaction dynamics. The TiO_2 nanobelts at large MF shows the rate constant k of 0.036 min^{-1} which is 3.6 times of that of TiO_2 nanobelts at NMF (0.01 min^{-1}), 1.38 times of that of TiO_2 nanobelts at small MF (0.026 min^{-1}) and 1.2 times of that of TiO_2 nanobelts at medium MF. Moreover, TiO_2 nanobelts at 900 rpm (one NdFeB magnet, T: 10 mm, D: 50 mm) in Fig S9 (b) shows the rate constant k of 0.038 min^{-1} which is 3.8 times of

that of TiO_2 nanobelts at NMF (0.01 min^{-1}), 1.26 times of that of TiO_2 nanobelts at 600 rpm (in the same magnetic field) (0.03 min^{-1}) and 1.22 times of that of TiO_2 nanobelts at 1200 rpm (0.031 min^{-1}).

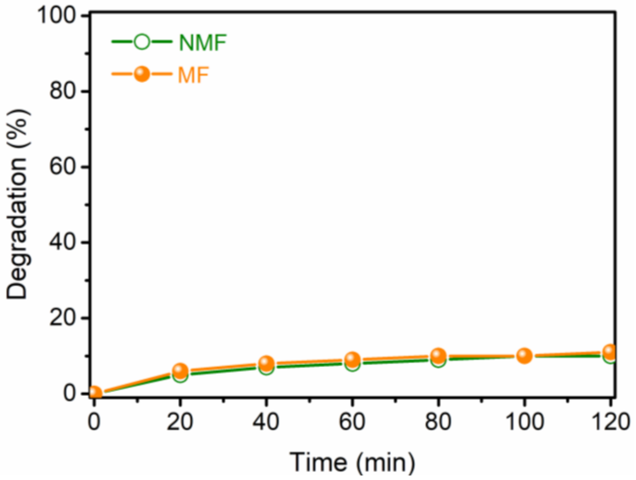

Figure S10. Photocatalytic degradation of MO at NMF or MF (medium MF 810 Gauss) in the presence of TiO_2 nanobelts under visible-light irradiation ($\lambda \geq 400$ nm).

The photocatalytic performance of TiO_2 nanobelts in the visible light at external MF and NMF conditions are almost the same as shown in Figure S10. It indicates that the stirring in magnetic field almost does not broaden the visible light (300W Xe lamp, 100mw cm^{-2}, λ>400nm) absorption wave band for more charge generation, which means that without the carrier produced by photoelectric conversion, Lorentz force could not work for enhancement of photocatalysis process.

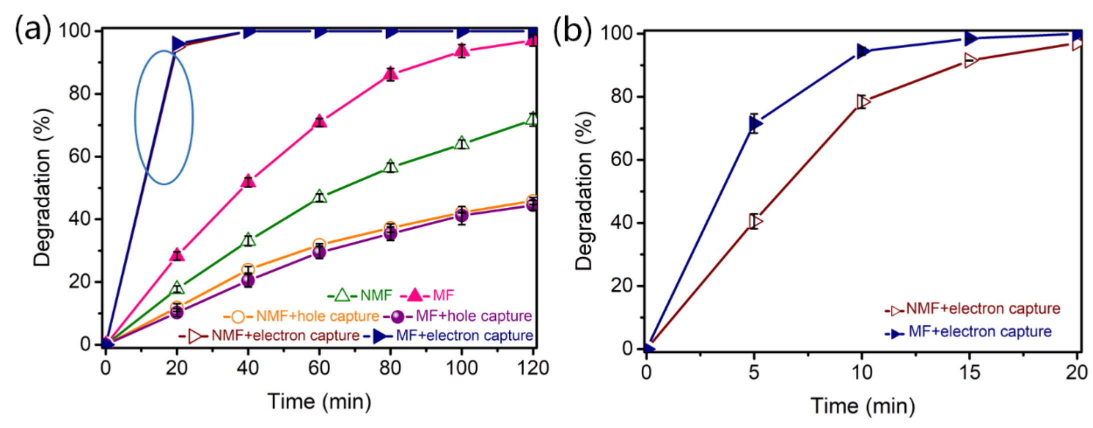

Figure S11. (a) Photocatalytic degradation of MO with different capture agents at NMF or MF in the presence of TiO_2 nanobelts under 20 mW cm^{-2} UV light irradiation (b) A more elaborate photocatalytic degradation of MO with electron capture at NMF or MF.

To further understand the mechanism of Lorentz force acted on the separation of charge carriers. Figure S11 shows the photocatalytic activities of TiO_2 nanobelts at the medium MF and NMF when the hole capture or electron capture were added in the photocatalytic system. As shown in Figure S11 (a), with the hole capture agent, the degradation rates of MO were 45% and 46% at the MF and NMF after degradation for 2 h, respectively. This result indicates that the scarification of holes limits the photocatalytic degradation of MO, which confirms that the photo-generated holes play a major role in the photocatalytic degradation of MO. In contrast, the photocatalytic performance was significantly improved with the adding of electron capture agent, realizing a complete degradation of MO in only 20 min. It indicates that more electrons were sacrificed resulting in more holes left for the MO degradation, which further confirms the importance of the charge separation for the improvement of photocatalysis. As shown in Figure S11 (b), within 20 min, the photocatalytic performance of TiO_2 nanobelts at MF was higher than that of NMF with the electron capture agent, which is consistent with better charges separation at MF. However, as is well known, electron capture has a quite strong

property to improve the charge separation. In this case, how did the MF further increase the separated holes?

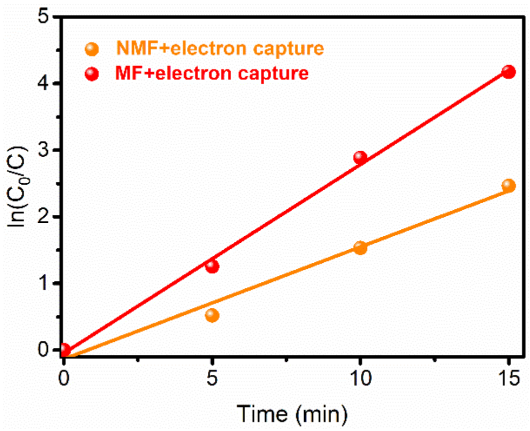

Figure S12. The ln (C_0/C) as a function of UV irradiation time in the presence of the TiO_2 nanobelts with electron capture at MF and NMF.

As shown in Figure S12, the photocatalytic performance of TiO_2 nanobelts at MF and NMF with the electron capture agent also follows the first-order reaction dynamics. The TiO_2 nanobelts at MF shows the rate constant k of 0.283 min^{-1} which is 1.68 times of that of TiO_2 nanobelts at NMF (0.01 min^{-1}) with the electron capture agent.

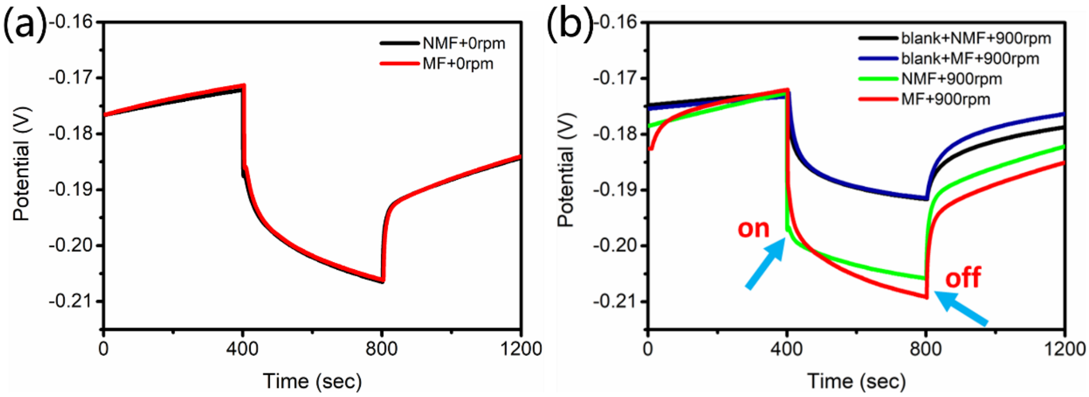

Figure S13. (a) Time dependence of open circuit potential of TiO_2 nanobelts under UV light irradiation and 0 rpm at MF and NMF. (b) Time dependence of open circuit potential of TiO_2 nanobelts and blank sample under UV light irradiation and 900 rpm at MF and NMF.

The open-circuit potential time curve was also measured with the light illumination on and off. When the light was on, there were an obvious response with 0.207 V open-circuit potential (OCP) at the 0 rpm at MF and NMF for both the samples of TiO_2 nanobelts as shown in Figure S13 (a). However, an enhancement of 0.003 V OCP can be observed when the speed of the rotate disc electrode was adjusted to 900 rpm at MF than that in the same condition at NMF. In addition, the OCP of blank sample has basically nothing changed at the 900 rpm at MF and NMF as shown in Figure S13 (b). This also proved that Lorentz force in magnetic field is the main reason to promote the separation of charge carrier from the other aspect.

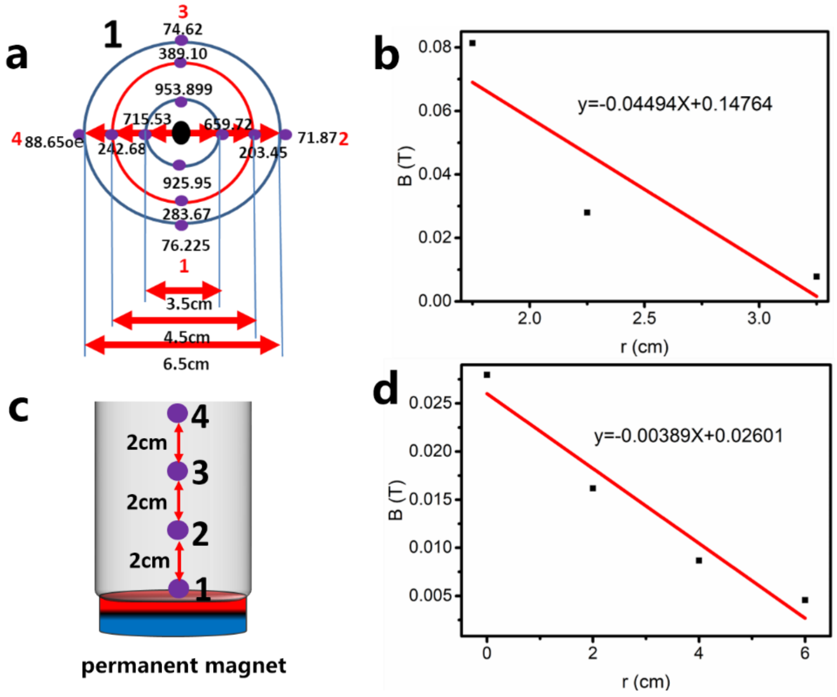

Figure S14. Taking (a) and (c) as medium magnetic field (one NdFeB magnet, thickness (T) 10mm, diameter (D) 50mm) templates, fitting the distribution of magnetic induction intensity in the direction of crosswise (b) and lengthways (d).

For the distribution of magnetic induction intensity in space, we chose the point 1 that perpendicular to the direction of magnetic field as the transverse starting point and the average distribution of magnetic field in this plane was fitted linearly as shown in Figure S14 (a-b). Meanwhile, Figure 14 (c-d) shows the distribution of magnetic fields fitted linearly in the direction perpendicular to the magnetic field and selected the plane center point with a horizontal diameter of 3.5 cm.

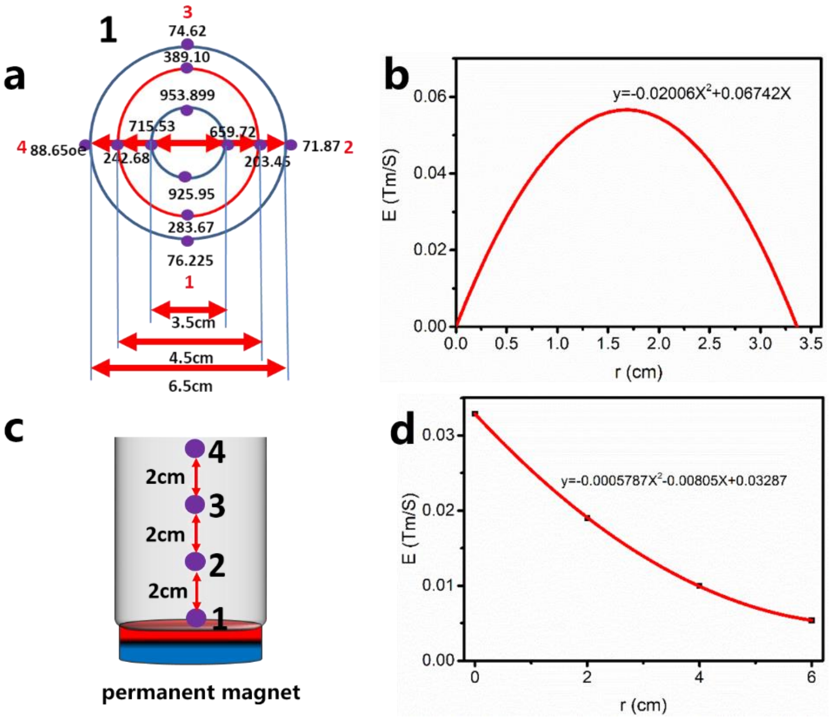

Figure S15. Taking (a) and (c) as medium magnetic field (one NdFeB magnet, thickness (T) 10mm, diameter (D) 50mm) templates, according to the formula: $\vec{F}_E=\vec{E}q$ fitting the distribution of electric field intensity in the direction of crosswise (b) and lengthways (d).

According to the fitted distribution of magnetic induction intensity in space in Figure S14, the transformed electric field formula was fitted at each point by the formula: $\vec{F}_E=\vec{E}q$ as shown in Figure S15 (b and d). According to the magnetic field distribution fitted in the longitudinal direction, we selected the electric field of the space point 4 cm above the vertical magnet and diameter 3.5 cm as the reference point (magnetic induction intensity is 0.01 T) as the applied electric field to research the first-principles calculation.
